# Canine Platelet Lysate Is Inferior to Fetal Bovine Serum for the Isolation and Propagation of Canine Adipose Tissue- and Bone Marrow-Derived Mesenchymal Stromal Cells

**DOI:** 10.1371/journal.pone.0136621

**Published:** 2015-09-09

**Authors:** Keith A. Russell, Thomas W. G. Gibson, Andrew Chong, Carmon Co, Thomas G. Koch

**Affiliations:** 1 Department of Biomedical Sciences, Ontario Veterinary College, University of Guelph, Guelph, Canada; 2 Clinical Studies, Ontario Veterinary College, University of Guelph, Guelph, Canada; 3 The Orthopaedic Research Lab, Aarhus University, Aarhus, Denmark; Wake Forest Institute for Regenerative Medicine, UNITED STATES

## Abstract

**Background:**

Mesenchymal stromal cells (MSC) are increasingly investigated for their clinical utility in dogs. Fetal bovine serum (FBS) is a common culture supplement used for canine MSC expansion. However, FBS content is variable, its clinical use carries risk of an immune response, and its cost is increasing due to global demand. Platelet lysate (PL) has proven to be a suitable alternative to FBS for expansion of human MSC.

**Hypothesis and Objectives:**

We hypothesized that canine adipose tissue (AT) and bone marrow (BM) MSC could be isolated and expanded equally in PL and FBS at conventionally-used concentrations with differentiation of these MSC unaffected by choice of supplement. Our objectives were to evaluate the use of canine PL in comparison with FBS at four stages: 1) isolation, 2) proliferation, 3) spontaneous differentiation, and 4) directed differentiation.

**Results:**

1) Medium with 10% PL was unable to isolate MSC. 2) MSC, initially isolated in FBS-supplemented media, followed a dose-dependent response with no significant difference between PL and FBS cultures at up to 20% (AT) or 30% (BM) enrichment. Beyond these respective peaks, proliferation fell in PL cultures only, while a continued dose-dependent proliferation response was noted in FBS cultures. 3) Further investigation indicated PL expansion culture was inducing spontaneous adipogenesis in concentrations as low as 10% and as early as 4 days in culture. 4) MSC isolated in FBS, but expanded in either FBS or PL, maintained ability to undergo directed adipogenesis and osteogenesis, but not chondrogenesis.

**Conclusions/Significance:**

Canine PL did not support establishment of MSC colonies from AT and BM, nor expansion of MSC, which appear to undergo spontaneous adipogenesis in response to PL exposure. *In vivo* studies are warranted to determine if concurrent use of MSC with any platelet-derived products such as platelet-rich plasma are associated with synergistic, neutral or antagonistic effects.

## Introduction

Over the last 15 years, increasing efforts have been put toward the innovation and optimization of stem cell-based therapies for conditions where currently there are poor or no treatments available. Mesenchymal stromal cells (MSC), in particular, have attracted attention owing to the cells’ ability to differentiate into various tissue lineages and their easy and ethical accessibility and expandability [1–3]. In canine studies, MSCs have been isolated, expanded, and extensively characterized from bone marrow (BM-MSC), adipose tissue (AT-MSC), and umbilical cord blood and their potential demonstrated for proliferation and for induced osteogenesis, adipogenesis, and chondrogenesis *in vitro* [4–6].

MSC are rare cells found at a concentration of less than 0.1% of marrow cells in human newborns—a concentration that decreases with patient age [7]. While adipose tissue is reported to yield a 500-fold increase in MSC per millilitre when compared to marrow, *ex vivo* expansion is still required before the amount and concentration necessary for clinical application is acquired [8,9].

Whether in human or canine research, a lack of standardized protocols is a key barrier to clinically safe and reliable MSC-based therapies [10]. One of the known causes of variability in current protocols is the inclusion of fetal bovine serum (FBS) in the culture medium used. FBS is typically added between 5% and 30% to supplement the basal medium DMEM or αMEM. In addition to the problem of undefined and variable content from batch to batch, there are risks of complications from bovine antigens [11–13]. Exploration into possible alternatives to FBS has been taking place for years opening up many promising avenues. Finding alternatives to FBS is becoming more pressing from a financial perspective as supply and demand issues have significantly increased the price of FBS [14].

Human platelet lysate (PL) has been extensively studied and repeatedly proven to outperform FBS as a supplement in expansion media [15–18]. PL is a concentrated solution of growth factors released from the platelet fraction by a freeze-thaw procedure. Pooled human PL has been found to be the most suitable to provide an alternative to FBS for clinical-scale expansion [11,12,17]. Factors within the PL include platelet-derived growth factor (PDGF), transforming growth factor-β (TGF-β), epidermal growth factor (EGF), and vascular endothelial growth factor (VEGF) [19].

Research into a FBS-free medium for the culture of canine MSC is extremely limited at this time. We hypothesized that canine AT- and BM-MSC could be isolated and expanded equally in PL and FBS at conventionally-used concentrations with differentiation of these MSC unaffected by choice of supplement.

## Materials and Methods

### Ethics statement

This study adhered to the guidelines by the University of Guelph Animal Care Committee with regard to the procedures of collection of canine adipose tissue and bone marrow samples. Collection of such tissue samples post-mortem and subsequent research conducted using specimens of this kind does not require review by the Animal Care Committee (falls under CCAC Category of Invasiveness A). The studies presented in this manuscript can be considered to have been conducted in accordance with the institutional ethics guidelines. Dogs were sacrificed for reasons unrelated to the studies prior to collection of adipose tissue and bone marrow.

### Platelet concentrate and platelet lysate

Platelet concentrate (PC) was collected by the Ontario Veterinary College Small Animal Clinic through methods previously described for reasons unrelated to this study [20]. After 5–7 days, canine PC was released for inclusion in this study, transferred to our lab, and processed immediately. PC was added to 50 mL tubes and centrifuged at 2000 *g* for 10 minutes. The pellets were resuspended in a minimal volume of platelet-poor plasma (PPP) and a complete blood count was run. Once the platelet concentration was known, the PC was diluted with platelet-poor plasma (PPP) to 1 × 10^6^ platelets/μL according to convention [21]. The PC was frozen to -80°C and thawed in a 37°C water bath to lyse the platelets. For use in medium, PL from 10 dogs were pooled, spun at 4000 *g* for 15 minutes, and filtered through a 0.22 μm filter (Millex-GP Filter Unit, Millipore, Etobicoke, Ontario) before addition.

### MSC isolation and expansion

Matched samples of bone marrow aspirate and adipose tissue were obtained from a total of 8 dogs. All dogs used weighed a minimum 30 kg, were of unknown age, and were to be euthanized for reasons unrelated to this study. The dogs were euthanized by intravenous injection of pentobarbital (Euthanyl Forte, 540mg/5 Kg, Biomeda-MTC Animal Health, Cambridge, Ontario) before bone marrow was extracted from the humerus with a 13 gauge Jamshidi needle (Kendall, Tyco Healthcare, Pointe-Claire, Quebec) and expelled into a 10 mL glass tube coated with heparin (Sandoz, Boucherville, Québec). Approximately 10 g of subcutaneous adipose tissue were surgically removed from the abdomen and placed in phosphate buffered saline (PBS, Roche, Indianapolis, Indiana) with 2% penicillin/streptomycin (Life Technologies, Grand Island, New York).

The adipose tissue was minced in a Petri dish and washed with equivalent volume of PBS with penicillin/streptomycin until the solution ran clear. The tissue was then digested in an equivalent volume of collagenase I (Sigma-Aldrich, St. Louis, Missouri) in PBS solution (1 mg/mL) for 60–180 minutes shaking at 200 rpm on a heat block set at 37°C. When digested, the solution was filtered twice through a 70 μm falcon strainer (BD, Franklin Lakes, New Jersey). The nucleated cell fraction was pelleted out by centrifugation at 400 *g* for 15 minutes before being resuspended in PBS. These steps were repeated 4 times with the volume separated into 2 tubes for the final spin to prepare for resuspension in the different treatment media. Bone marrow aspirates were plated directly in one of either FBS or PL treatment groups. All isolated cells were plated in 12 well plates with MSC expansion medium consisting of low glucose DMEM (Lonza, Walkersville, Maryland), 10% pooled FBS (Life Technologies, Grand Island, New York) or 10% pooled PL, 1% penicillin/streptomycin, and 1% L-glutamine (Lonza, Walkersville, Maryland). All cell cultures were incubated at 38°C in a humidified environment containing 5% CO₂.

All cultures had media completely replaced daily for the first 3 days to remove red blood cell contamination, after which they were changed every 2 days until colonies were large enough for passage. Colonies were considered large enough when they surpassed 0.75 mm in diameter. Cells were passaged by washing with PBS and incubating at 38°C in cell detachment solution (Accumax, Innovative Cell Technologies, San Diego, California) for 12 minutes before seeding at 5000 cells/cm². Only cultures that expanded to a minimum of 5 million cells were cryopreserved for further experiment.

### MSC proliferation

AT-MSC and BM-MSC isolated in FBS (n = 10) were thawed from cryopreservation and plated in MSC medium with 10% FBS. After 5 days, they were passaged and seeded at 5000 cells/cm² into 96 well plates again with 10% FBS medium. After cells attached (overnight), all medium was removed and replaced by either FBS or PL treatment medium at concentrations 5, 10, 20, 30, 40, 50 or 60% in quadruplicate wells. Proliferation was analyzed by resazurin assay (Sigma-Aldrich, St. Louis, Missouri) after removal of all treatment media, addition of PBS with 10% resazurin (i.e. no FBS or PL), and incubation for 4 hours at 37°C. Plates were read at 585 nm using an excitation wavelength of 555 nm according to the manufacturer's directions by plate reader (SpectraMax i3 Multi-Mode microplate reader, Molecular Devices, Sunnydale, California).

### Spontaneous differentiation

The same AT-MSC and BM-MSC cultures used for the proliferation assays were also seeded at 5000 cells/cm² into 48 well plates for spontaneous differentiation testing by tri-lineage staining. Adipogenesis was assessed by Oil Red O (Sigma-Aldrich, St. Louis, Missouri) staining of lipids. Osteogenesis and chondrogenesis were assessed using a von Kossa staining protocol, which involved the addition of 1% silver nitrate (Sigma-Aldrich, St. Louis, Missouri) and exposure to UV light for 60 min, before counter-staining with Toluidine Blue (Sigma-Aldrich, St. Louis, Missouri). A plate from each culture was stained at days 4, 8, and 12 after treatments were added.

Parallel 6 well plates of the same cultures were grown and analysed for adipogenic mRNA expression (Table 1) using primers previously described [5, 22]. Cells were scraped on day 12 and flash frozen in lysis buffer for subsequent RNA extraction (RNeasy kit, Qiagen, Hilden, Germany). cDNA was synthesized from 500 ng RNA using High Capacity cDNA Reverse Transcription Kit (Life Technologies, Grand Island, New York) following manufacturers' instructions. PCR reactions were performed using PerfeCta SYBR Green FastMix, ROX (Quanta BioScience, Gaithersburg, Maryland) with an Applied Biosystems 7300 Real Time PCR system. Data were analyzed with the 2-ΔΔCT method. Gene expression fold-change is presented as the PL-treated cultures relative to the FBS-treated cultures with GAPDH as reference gene.

**Table 1:** Primers used in qPCR.

Gene	Forward 5' to 3'	Reverse 5' to 3'	Reference
CEBPA	AGTCAAGAAGTCGGTGGACAAG	GCGGTCATTGTCACTGGTGAG	[5]
FABP4	ATCAGTGTAAACGGGGATGTG	GACTTTTCTGTCATCCGCAGTA	[5]
Leptin	CTATCTGTCCTGTGTTGAAGCTG	GTGTGTGAAATGTCATTGATCCTG	[5]
LPL	ACACATTCACAAGAGGGTCACC	CTCTGCAATCACACGGATGGC	[5]
PPARγ2	ACACGATGCTGGCGTCCTTGATG	TGGCTCCATGAAGTCACCAAAGG	[5]
GAPDH	TGTCCCCACCCCCAATGTATC	CTCCGATGCCTGCTTCACTACCTT	[22]

### Directed differentiation

Trilineage differentiation was performed through chemical induction into adipocytes, osteocytes, and chondrocytes in parallel with non-induced controls. For adipogenesis and osteogenesis, 6 AT- and 4 BM-MSC isolated in 10% FBS and expanded between 3 and 5 passages were seeded at 5000 cells/cm² and grown to 90% confluence in 10% PL or 10% FBS expansion medium detailed above. To induce adipogenesis, the cells were cultured for 14 days in DMEM-LG with 1 μM dexamethasone (Sigma-Aldrich, St. Louis, Missouri), 0.5 mM 3-isobutyl-1- methyl-xanthine (Sigma-Aldrich, St. Louis, Missouri), 10 μg/mL recombinant human (rh) insulin (Sigma-Aldrich, St. Louis, Missouri), 0.2 mM indomethacin (Sigma-Aldrich, St. Louis, Missouri), 15% rabbit serum (Sigma-Aldrich, St. Louis, Missouri), 1% L-glutamine, and 1% ABAM. To induce osteogenesis, the cells were cultured for 14 days in DMEM-LG with 0.1 μM dexamethasone, 10 mM glycerol 2-phosphate, 0.05 mM ascorbic acid, 10% FBS, 1% L-glutamine, and 1% ABAM (Life Technologies, Grand Island, New York). For chondrogenesis, 250,000 cells per well in a 96-well plate were spun down (200 *g*, 10 min, RT) to form a pellet and cultured for 14 days in DMEM-HG (Lonza, Walkersville, Maryland), 0.1 μM dexamethasone, 0.1 mg/mL ascorbic acid (Sigma-Aldrich, St. Louis, Missouri), 10 ng/mL TGF-β3 (R&D Systems, Minneapolis, Minnesota), 200 mM Glutamax (Life Technologies, Grand Island, New York), 10 mg proline (Sigma-Aldrich, St. Louis, Missouri), 40 μg/mL ascorbic acid, 100 mM sodium pyruvate (Life Technologies, Grand Island, New York), 1% ITS (Life Technologies, Grand Island, New York), 1% L-glutamine, and 1% ABAM. Adipogenesis was assessed by Oil Red O staining of lipids and osteogenesis was assessed using Alizarin Red S (Sigma-Aldrich, St. Louis, Missouri) stain for calcium as previously reported [23]. Chondrogenesis was histologically evaluated using Toluidine Blue stain for glycosaminoglycan content as previously reported [24].

### Data analysis

All data were analyzed using R statistical software (version 3.1.3., The R Foundation for Statistical Computing, Vienna, Austria). For the proliferation data, results were modelled as a 3-factor factorial in a randomized complete block design (RCBD) treating dog as a blocking factor. A log transform of the plate readings minus 7% more than the smallest reading (due to some negative values) was performed for a more normal distribution. Least squares means were determined and back-transformed for readability. For the qPCR data, results were modelled as a 3-factor factorial in a RCBD treating dog as a blocking factor. MSC source was removed from the model as it showed no evidence of effect (p = 0.74). We formally tested residuals for normality and plotted them against the predicted values and explanatory variables to assess ANOVA assumptions and to look for unequal variance and outliers. Based on this analysis, the data were fundamentally normal except for outliers, which were kept in the data set. Least squares means were determined and converted to fold-difference by using base 2 to the power of the-ΔΔCT. Data are presented as mean ± confidence interval. Statistical difference was assessed at P<0.05.

## Results

### MSC isolation and expansion

**Fig 1:**
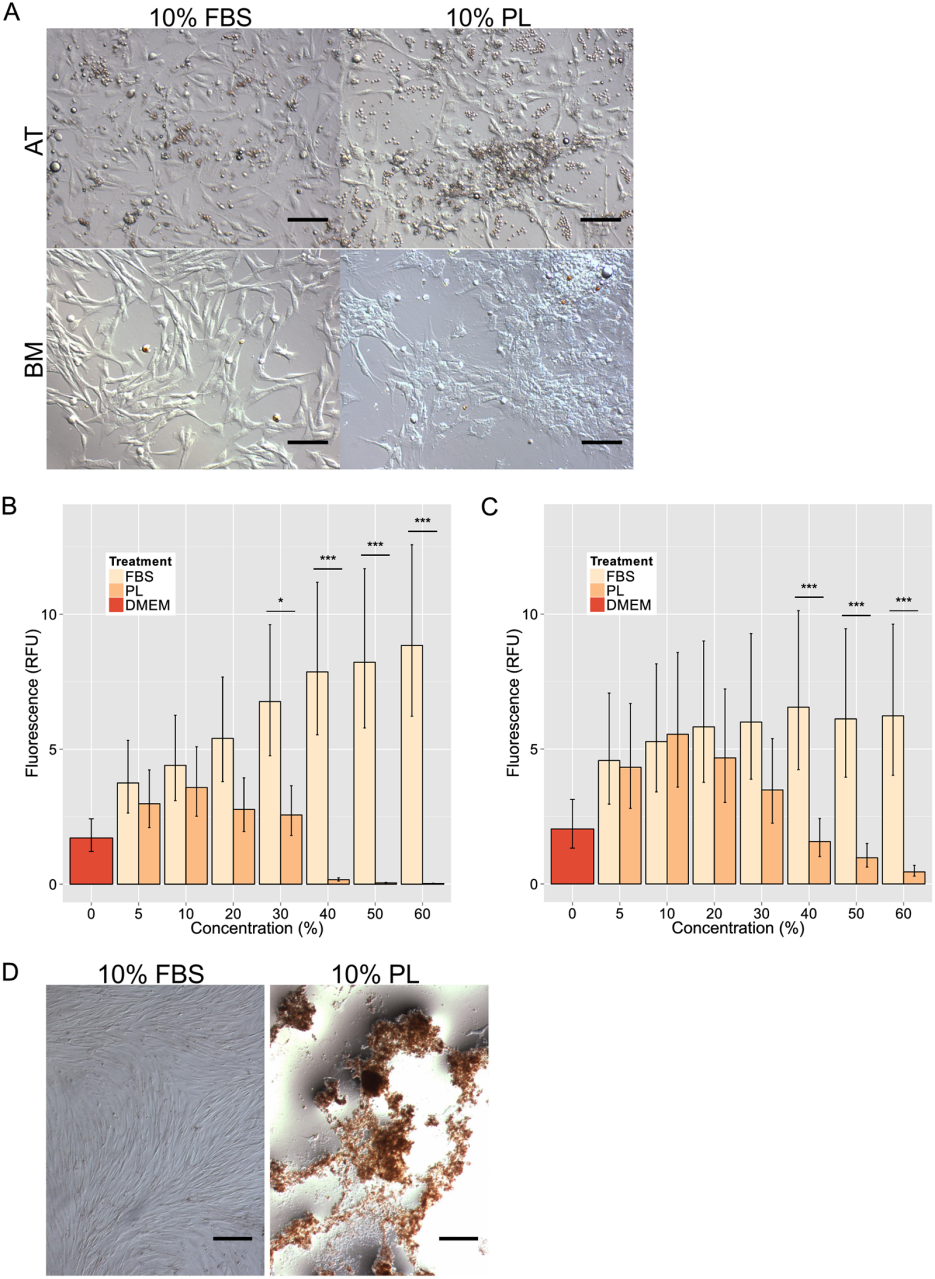
MSC isolation and proliferation in PL vs. FBS culture. (A) Primary canine adipose tissue (AT)- and bone marrow (BM)-derived mesenchymal stromal cell (MSC) colonies isolated in 10% fetal bovine serum (FBS) or 10% platelet lysate (PL). Scale bar = 50 μm. (B) Proliferation assays (resazurin) of AT-MSC (n = 6) and (C) BM-MSC (n = 4) in FBS- or PL-enriched medium from 5% to 60%. Base medium (DMEM) was used as a negative control. (*P<0.05, ***P<0.001; error bars = CI.) D) Long-term effect of 10% FBS and 10% PL expansion media on MSC over period of 21 days without passage. Cultures stained with Alizarin Red S. Scale bar = 250 μm.

Isolation success was based not only on colony formation, but also each population's ability to expand to sufficient numbers of 5 million cells, as outlined above, within 4 passages for cryopreservation. Based on these criteria, while 7 out of 16 populations cultured in PL-supplemented medium formed colonies, growth of these colonies arrested before sufficient numbers could accumulate (Table 2). In contrast, 14 of the 16 FBS-supplemented cultures met isolation criteria. Elements unrelated to typical MSC morphology can be seen among the PL-supplemented colonies compared to their FBS-supplemented counterparts (Fig 1A).

**Table 2:** Isolation success of canine AT- and BM-MSC in 10% FBS or 10% PL.

Source	Treatment	Colony formation	Expanded to 5e6 cells
Adipose tissue	FBS	8/8	8/8
	PL	3/8	0/3
Bone marrow	FBS	8/8	6/8
	PL	4/8	0/4

### MSC proliferation

AT-MSC (n = 6) and BM-MSC (n = 4) established in the presence of FBS, but expanded in PL exhibited maximal proliferation at 10% PL (Fig 1B and 1C). In contrast, the same MSC cultures expanded in FBS exhibited increased proliferation rates with increased FBS concentration until a plateau was reached. There were no significant differences between the treatment groups up to 20% in the AT-MSC (Fig 1B) and 30% in the BM-MSC (Fig 1C). Significant differences were found at 30% in the AT-MSC (P = 0.043) and in both AT-MSC and BM-MSC at 40%, 50%, and 60% concentrations (P < 0.001).

Morphological differences can be seen in cells cultured for 21 days without passage with FBS-cultured cells maintaining tight spindle shapes and PL-cultured cells clumping together and detaching showing little resemblance to MSC (Fig 1D).

### Spontaneous differentiation

**Fig 2:**
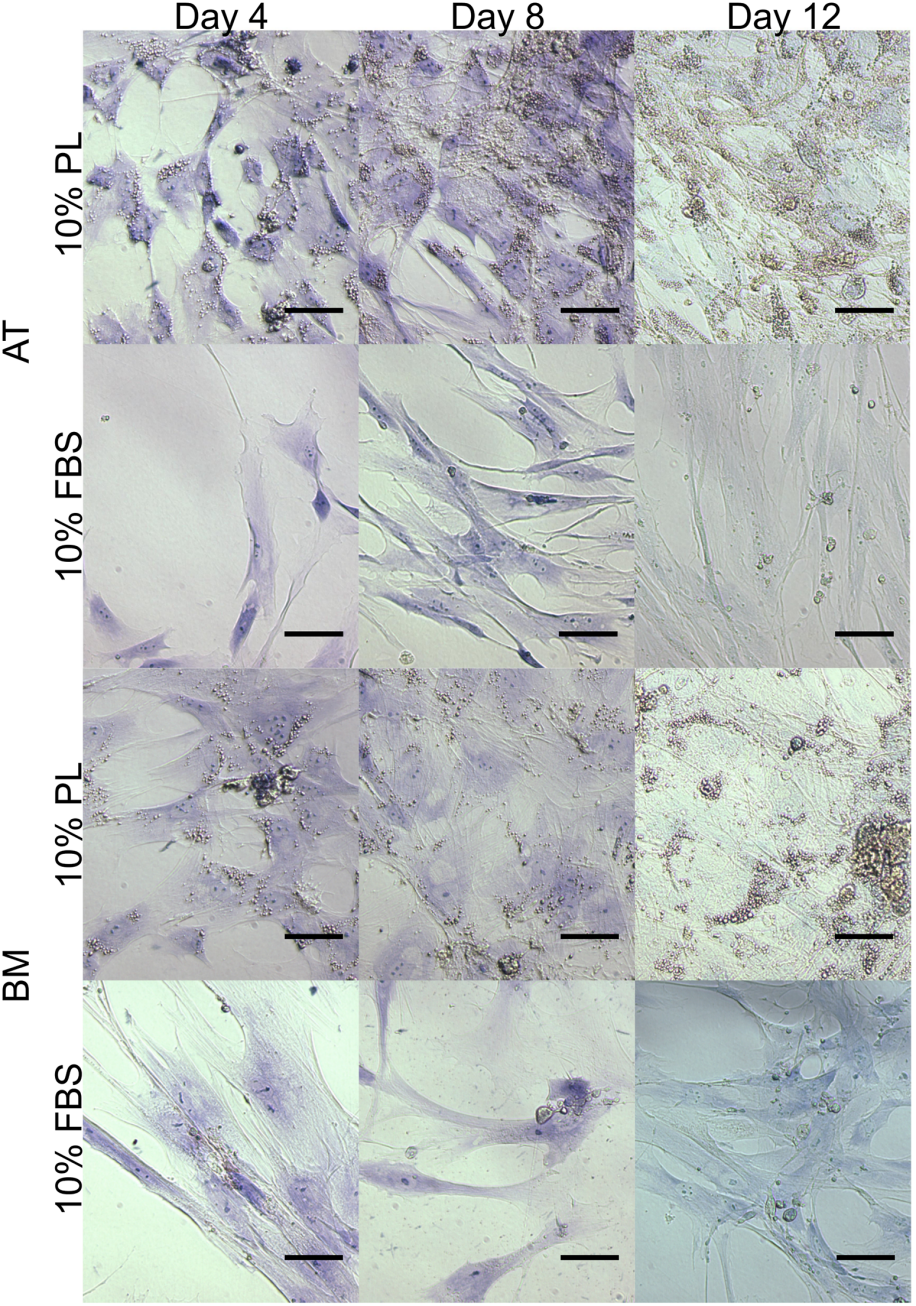
Short-term effect of MSC in PL vs. FBS culture. Effect of 10% platelet lysate (PL) and 10% fetal bovine serum (FBS) expansion media on canine adipose tissue (AT)- and bone marrow (BM)-derived mesenchymal stromal cells over 12 days. Cultures stained with the von Kossa protocol and Toluidine Blue for early markers of osteogenesis and chondrogenesis respectively. Scale bar = 50 μm.

**Fig 3:**
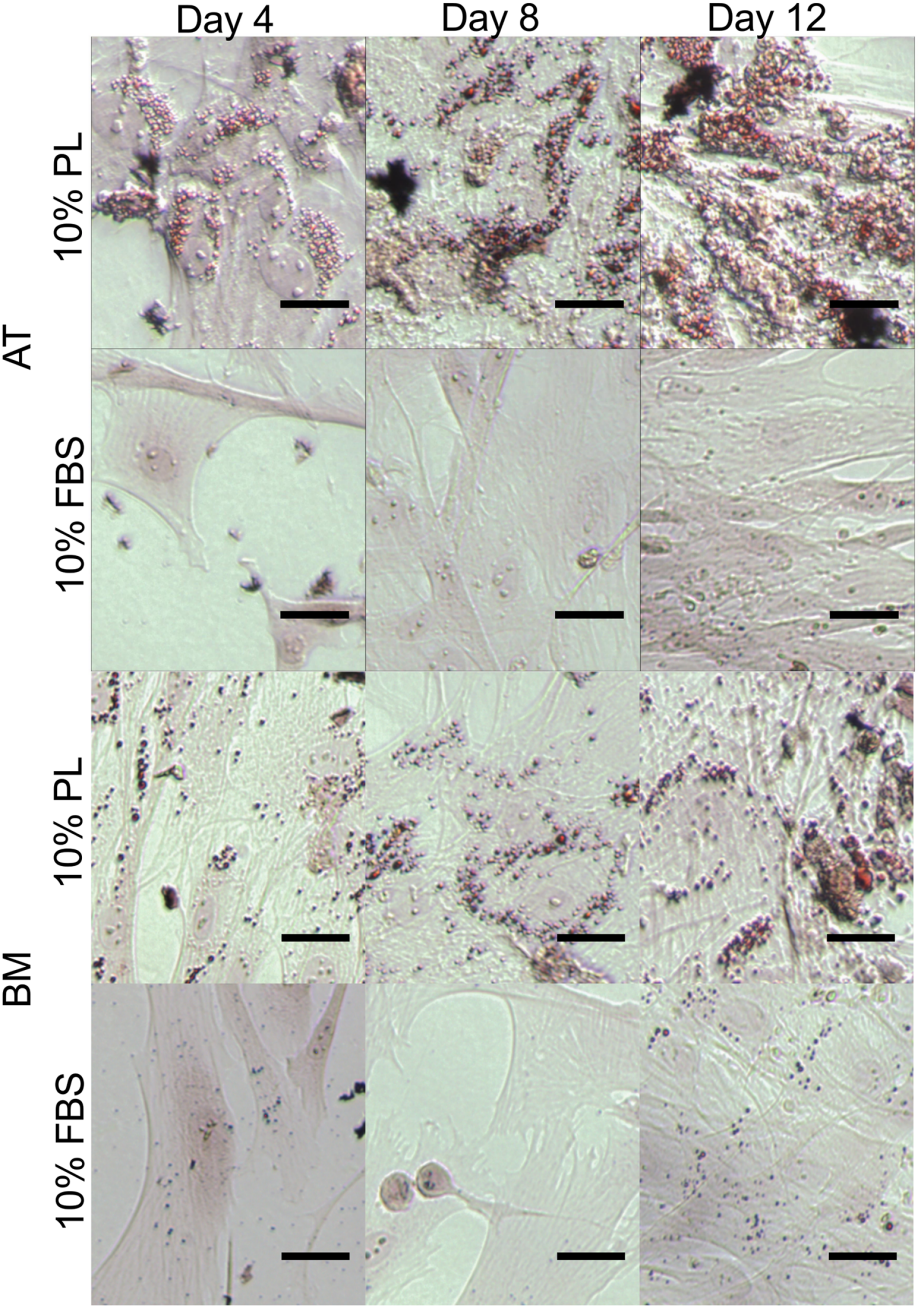
Short-term effect of MSC in PL vs. FBS culture. Effect of 10% platelet lysate (PL) and 10% fetal bovine serum (FBS) expansion media on canine adipose tissue (AT)- and bone marrow (BM)-derived mesenchymal stromal cells (MSC) over 12 days. Cultures stained with Oil Red O for early markers of adipogenesis. Lipid droplets seen forming in the PL cultures only. Scale bar = 250 μm.

Common to AT- and BM-MSC, both FBS and PL treatment groups stained negative for chondrogenesis and osteogenesis when combining Toluidine Blue and von Kossa after 4, 8, and 12 days in culture (Fig 2). When stained for adipogenesis with Oil Red O, lipid droplets can be seen forming only in the PL-treated cells as early as day 4 and progressively more in days 8 and 12 (Fig 3).

**Fig 4:**
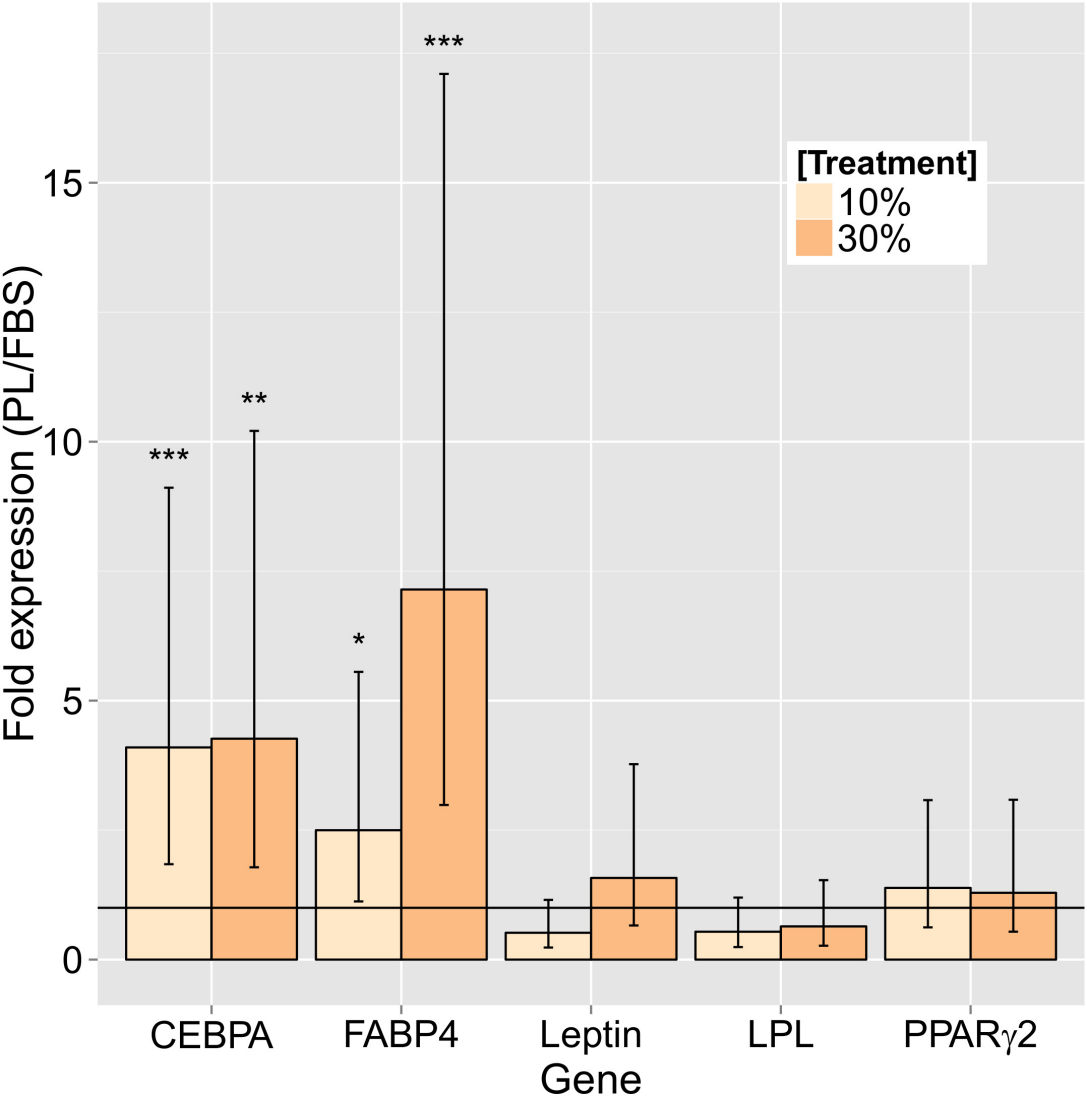
Adipogenic gene expression of MSC in short-term culture of PL vs. FBS culture. Fold difference adipogenesis marker expression of canine adipose tissue- and bone marrow-derived mesenchymal stromal cells cultured in fetal bovine serum (FBS)- or platelet lysate (PL)-enriched medium at 10% and 30% after 12 days. (*P<0.05, **P<0.01, ***P<0.001; error bars = CI.)

Adipogenic mRNA expression of *CEBPA* and *FABP4* was up-regulated in both 10% and 30% concentrations of PL-treated 12-day cultures when compared to the FBS-treated cultures (Fig 4). For *CEBPA*, there was close to 4-fold expression in both 10% (3.86, P < 0.001) and 30% (3.92, P = 0.002) PL-treated MSC. For *FABP4*, there was 2.35-fold expression at 10% PL (P = 0.026) and 6.56-fold expression at 30% PL (P < 0.001) compared to the same concentration FBS. There were no significant differences found between the treatments for the expression of *Leptin*, *LPL*, or *PPARγ2*.

### Directed differentiation

**Fig 5:**
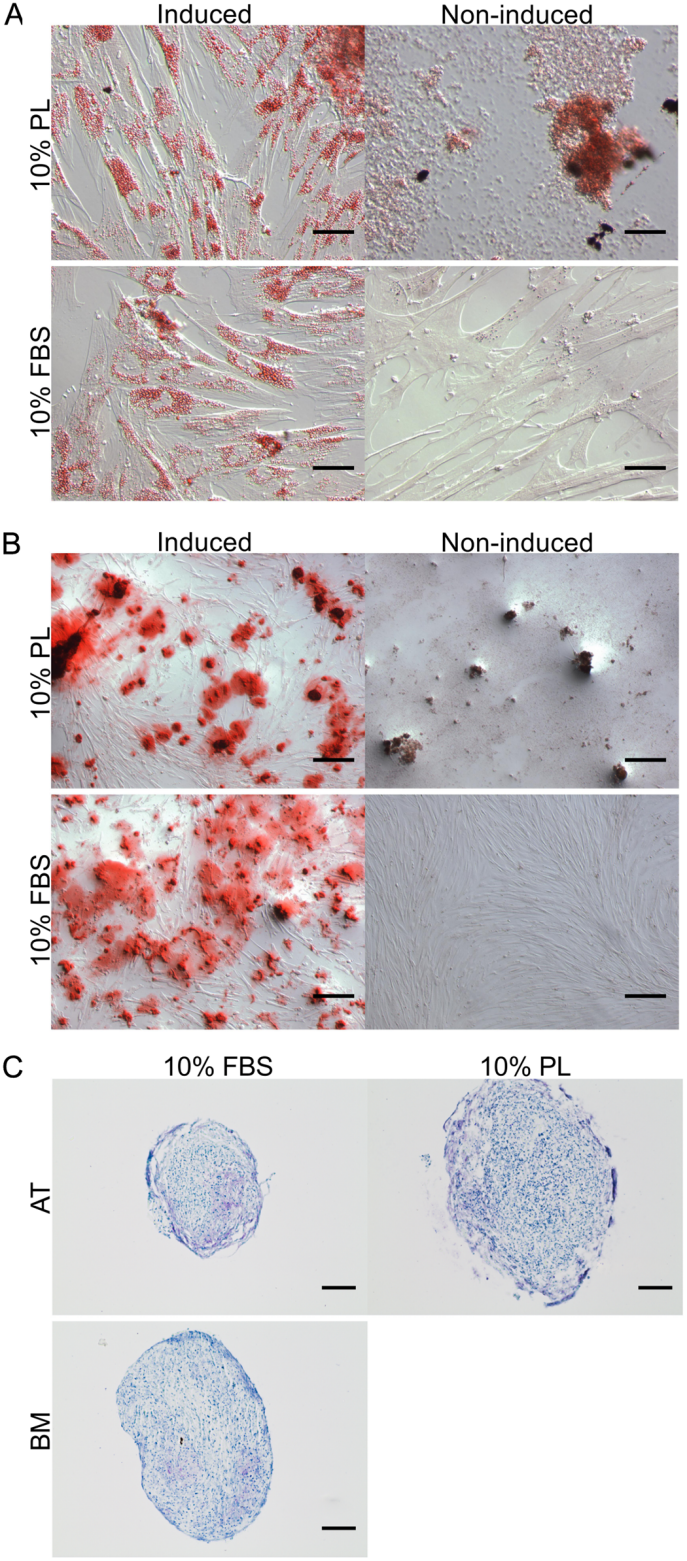
Directed differentiation of canine PL- and FBS-cultured MSC. Trilineage differentiation of canine mesenchymal stromal cells (MSC) previously expanded in 10% platelet lysate (PL) or fetal bovine serum (FBS). (A) Adipogenic potential of adipose tissue (AT)-derived (MSC) was assessed with Oil Red O staining after 14 days in induction medium. Adjusted for brightness. Scale bar = 50 μm. (B) Osteogenic potential of AT-MSC was assessed with Alizarin Red S staining after 14 days in induction medium. Adjusted for brightness. Scale bar = 250 μm. (C) Chondrogenic potential of AT- and bone marrow (BM)-MSC was assessed with Toluidine blue staining after 21 days in induction medium. Control pellets cultured in expansion medium did not remain intact through histological processing. Adjusted for contrast. Scale bar = 100 μm. No BM-MSC cultured in PL expanded sufficiently for adipogenesis or osteogenesis.

A minimum number of 4.6 million cells were necessary for trilineage (adipogenesis, osteogenesis, and chondrogenesis) differentiation studies. While all 10 FBS cultures (6 AT- and 4 BM-MSC) yielded enough cells, only 3/10 PL cultures (3 AT-MSC) expanded sufficiently for trilineage differentiation. After 14 days in induction medium, MSC previously grown in either PL or FBS stained positive for adipogenesis (Fig 5A) and osteogenesis (Fig 5B). Chondrogenesis was unsuccessful under the conditions provided in this study of pellet cultures from both AT- and BM-MSC and both FBS and PL treatment groups (Fig 5C).

## Discussion

Our results show PL performing inadequately in the key areas of MSC isolation and long-term expansion. However, PL may yet prove feasible as an alternative to FBS in certain areas of canine MSC culture such as in transition medium just prior to clinical use or differentiation.

While colonies did emerge from digested adipose tissue and bone marrow cultured in isolation medium with 10% PL, none of these colonies were robust enough to yield numbers high enough for long-term cell banking. Almost all (14 out of 16) MSC populations isolated in FBS grew to sufficient number for cryopreservation. Distinct morphological differences were seen in the primary colonies especially in the BM primary colonies (Fig 1A). It may be that other concentrations of PL could have improved on isolation success as only a concentration of 10% was used in the PL isolation medium.

Similar to our recent findings with equine cord blood-derived MSC [25], PL supported canine AT- and BM-MSC proliferation similarly to FBS only at lower concentrations up to 20% (AT-MSC) or 30% (BM-MSC) before it became detrimental to cellular growth (Fig 1B and 1C). When left long-term in PL culture for 21 days without passage, MSC were prone to clump together and lift off the plate, whereas MSC for the same length of time in FBS retained a tight formation of spindle-shaped cells (Fig 1D).

Spontaneous differentiation down the adipogenic line may offer an explanation to these findings. Adipogenic staining of MSC cultured in as low as 10% PL and for as briefly as 4 days show pervasive evidence of lipid droplet formation when compared to those grown in 10% FBS (Fig 3). Lipid droplets were seen to accumulate increasingly over time only in the PL cultures (Fig 3). Stronger mRNA expression of *CEBPA* and *FABP4* in the PL cultures was also found to support these findings (Fig 4). Expression of these two markers are known to increase progressively through adipogenesis [26].

Directed differentiation appears unaffected by prior choice of expansion medium supplement. Both adipogenic and osteogenic induction show a comparable level of staining after 14 days with Oil Red O and Alizarin Red S respectively (Fig 5A and 5B). Chondrogenesis via pellet culture was also attempted using the lab-established protocol used for equine MSC [23,27,28]. However, this protocol did not produce convincing histological evidence of chondrogenesis with MSC grown in either PL or FBS (Fig 5C). Similar difficulties inducing chondrogenesis in canine MSC have been reported [29].

Overall, these findings were unexpected since PL is being increasingly used to support the propagation of various human MSCs [15–18]. We noticed a similar dose-dependent effect of PL on equine cord blood-derived MSC [25]. Complement-induced lysis by the PL was ruled out due to diminished proliferation of MSC treated with heat-inactivated PL (data not shown).

Taken together, these results raise some concern about clinical use of platelet-derived products like platelet-rich plasma (PRP) with MSC. PRP should not be considered a neutral carrier solution for MSC. Activated platelets appear to compel adipogenic differentiation of MSC even when dilute. Therefore, co-culture or co-injection of these elements may significantly limit the regenerative capacity of the MSC. Further study into *in vivo* effects of MSC in combination with platelet-derived products is justified.

## Supporting Information

S1 Dataset Raw proliferation data. (CSV)

S2 Dataset Raw qPCR data. (CSV)
